# Evaluating the effect of type 2 diabetes mellitus on CYP450 enzymes and P-gp activities, before and after glycemic control: A protocol for a case–control pharmacokinetic study

**DOI:** 10.1016/j.mex.2020.100853

**Published:** 2020-03-07

**Authors:** Navid Neyshaburinezhad, Mohammadreza Rouini, Nooshin Shirzad, Alireza Esteghamati, Manouchehr Nakhjavani, Soha Namazi, Yalda H. Ardakani

**Affiliations:** a Biopharmaceutics and Pharmacokinetic Division, Department of Pharmaceutics, Faculty of Pharmacy, Tehran University of Medical Sciences, Tehran, Iran; b Endocrinology and Metabolism Research Center (EMRC), Vali-Asr Hospital, School of Medicine, Tehran University of Medical Sciences, Tehran, Iran; c Department of Clinical Pharmacy, Faculty of Pharmacy, Tehran University of Medical Sciences, Tehran, Iran

**Keywords:** Diabetes mellitus, Cytochrome P450, Metabolism, Personalized medicine

## Abstract

Cytochrome P450s (CYP450) family is one of the most critical factors in the metabolism process. Hence, the present study aims to characterize the activity of CYP1A2, CYP2B6, CYP2C9, CYP2C19, CYP2D6, CYP3A4/5, and P-glycoprotein (P-gp) pump in patients with type 2 diabetes (T2DM). This characterization was performed before and after good glycemic control versus non-diabetic subjects following the administration of a substrate probe drug cocktail. This single-center clinical study proposes the characterization of T2DM impacts on major CYP450 drug-metabolizing enzyme and P-glycoprotein (P-gp) activities. The main propose of the present study is evaluating any alternation in major CYP450 enzymes and P-gp activities in patients with T2DM, before (A1C>7%) and after (A1C≤7%) good glycemic control along with comparing the activities versus non-diabetic subjects. The phenotypes will be assessed following the oral administration of a drug cocktail containing caffeine (CYP1A2), bupropion (CYP2B6), flurbiprofen (CYP2C9), omeprazole (CYP2C19), dextromethorphan (CYP2D6), midazolam (CYP3A4/5), and fexofenadine (P-gp) as probe substrates. Furthermore, the influence of variables such as glycemia, genetic polymorphisms, and inflammation on the metabolism process will be evaluated. The first patient has entered the study in Dec 2018.

## Graphical abstract

**Figure:**
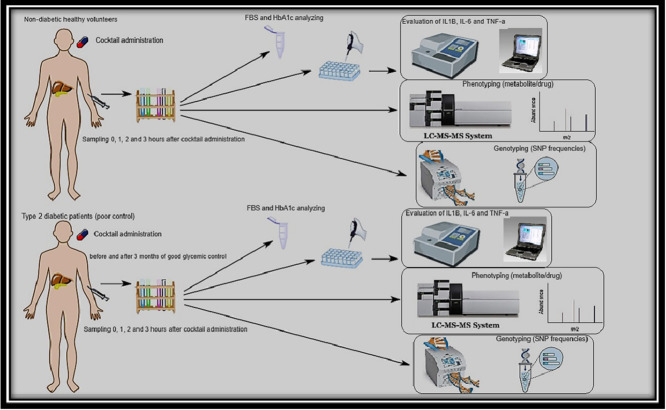

**Table:** Specifications Table

Subject area	Pharmacology, toxicology and pharmaceutical science
More specific subject area:	Personalized medicine and Phenoconversion of cytochrome P450 enzymes in patients with type 2 diabetes
Protocol name:	Evaluation the effect of type 2 diabetes mellitus on CYP450 enzymes and Pgp activities, before and after glycemic control: a protocol for a case-control pharmacokinetic study
Reagents/tools:	PCR method for genotype analysis
	LC-MS/MS for phenotype analysis
	Cocktail probe drugs
Experimental design:	This study is an open-label, non-randomized, two-armed (Poor controlled T2D patients and healthy volunteers), single-center explorative pharmacokinetic research
Trial registration:	Approval was obtained from the Tehran University of Medical Sciences
Ethics:	Ethics Review Board (Tehran, Iran) approval ID: IR.TUMS.TIPS.REC.1397.083)
Value of the Protocol	• Personalized medicine associated with targeted therapeutic and diagnostic concepts, was proposed to improve the efficacy and safety of health interventions
	• Cytochrome P450s (CYP450) family is one of the most critical factors in the metabolism process and prediction of drugs efficacy and safety of health interventions
	• Hence, the present study aims to characterize the activity of CYP1A2, CYP2B6, CYP2C9, CYP2C19, CYP2D6, CYP3A4/5, and P-glycoprotein (P-gp) pump in patients with type 2 diabetes (T2DM)

## Introduction

Personalized medicine associated with targeted therapeutic and diagnostic concepts, was proposed to improve the efficacy and safety of health interventions [1]. The relation between human drug-metabolizing enzymes and personalized medicine has become a hot topic according to the recent evolutions of the Human Genome Project [2]. Cytochrome P450 (CYP) superfamily plays an undeniable role in the biotransformation of almost 90% of currently used drugs^3^, other xenobiotics^4,^ and even endogenous compounds^5^ among all drug-metabolizing enzymes. The genotype variability in the pharmacokinetics of some medicines is believed to account for a considerable fraction of inter-individual deviations in corresponding clinical responses due to the activities of most drug-metabolizing enzymes (DMEs) are affected by genetic polymorphisms, [3]. Therefore, a large number of studies have been focused on the genotypes of DMEs to provide a specific and safe genotype-based dosing regimen based on the hypothesis exclusively on which genotype is predictive of DME phenotype and the clinical outcomes [4].

In addition, enzyme activities can be regulated by genetics and some extrinsic factors, e.g., a reduction in CYP3A and CYP2C activities due to elevation in pro-inflammatory [5]. It is believed that acute or chronic diseases, could stimulate the inflammatory processes leading to alter the expression of some CYPs and causing genotype-phenotype mismatches, as an essential environmental factor [6]. Therefore, emphasis on correlating the DME genotype of patients with clinical outcomes could carry a crucial risk that should be considered. Thus, entitled is one of the most implications as “phenoconversion,” in which an extensive genotypic metabolizer (EM) shows the DME phenotype of a Poor Metabolizer (PM) [6,7]. Consequently, it could be assumed that diabetes may act as an underlying environmental factor affecting the activity of CYP450 enzymes. Furthermore, it may lead to an erroneous prediction of the potential clinical response of administered medications, especially in the case of prodrugs and narrow therapeutic index medications.

In this regard, the effects of various diseases have been investigated in several animal studies, including diabetes, on the expression and activity of oxidative enzymes [8], [9], [10], [11].

Although animal models can provide valuable information about the possible effects of diabetes on the metabolic enzyme activity, it is difficult to evaluate the impact of disease type and state in these models; furthermore, the diabetes induction method can affect the interpretation of final results.

Most investigations have focused only on the impact of diabetes on the expression of oxidative enzymes, regardless of changes in their activities (phenotypes). In addition, in almost all studies, only one of the CYP enzymes have been investigated, and there are a few comprehensive investigations on the modulation of CYP450 activities in diabetes.

In this field, limited human studies are available, despite the observed changes in diabetic animal models [12].

Recently, the impact of type 2 diabetes (T2D) and its associated inflammatory processes on CYP450 metabolic activities were assessed in diabetic and non-diabetic volunteers after a single oral administration of a probe drug cocktail [13]*.* The result of this study indicated the modulation of CYP450 activities associated with diabetes in an isoform-specific manner [13,14].

Furthermore, pieces of evidence demonstrated that the CYP regulation pattern differs depending on disease type and state, and related proinflammatory cytokines, which are the major mediators of these effects. Thus, investigating the influence of various characteristics and variables of diabetes of disease state, including blood glucose levels and duration of disease. In addition, the duration of receiving treatments (good vs. poor control) and the levels of inflammatory cytokines on the metabolic enzyme activities should be considered.

The reduction in inflammatory cytokines of diabetes is expected after receiving appropriate treatments and glycemic control. Hence, the primary goal of the present study is to evaluate the modulation pattern in CYP450 phenotypes in diabetes patients. This evaluation was performed before and after three months of glycemic control as a pre-post study design using a multi-drug cocktail with reduced sampling frequency. Moreover, the investigators tend to examine the relationship between the inflammatory cytokines and the prevalence of “phenoconversion” before and after glycemic control. The design of this study minimized inter-individual variations to provide a better explanation for the impact of diabetes on CYP activities. Also, this design improved the concept of personalized medicine by helping to judge the necessity of dose adjustment during the medicinal treatment due to altered metabolic activity.

Further, CYP450 phenotypes will be compared with those of the healthy subjects (non-diabetic) and diabetes patients before and after receiving any treatment for glycemic control. They were considering a few human studies on the impact of diabetes in altering the function of these enzymes.

## Methods and analysis

### Study design

This study is an open-label, non-randomized, two-armed (Poor controlled T2D patients and healthy volunteers), and single-center explorative pharmacokinetic research. In this study, the effect of T2D on major CYP450 activities was investigated by focusing on the impact of glycemic control on the CYP phenotype alteration pattern.

The project is consists of two key clinical phases. In the first step, the pattern of changes in the phenotype of six major CYP enzymes and their relationship with the level of principal proinflammatory cytokines (IL-1β, IL-6, and TNF-α) in patients with type 2 diabetes will be examined. This examination was performed before and after adequate glycemic control as a primary endpoint by evaluating the “phenoconversion” approach.

For this purpose, a total of 42 adolescent patients of both genders will be enrolled in the first phase following approval from the Ethics Review Board and based on literature review and statistical calculations.

Therefore, the phenotypes will be assessed by calculating metabolic ratios using the oral administration of well-characterized low-dose probe cocktail (GENEVA cocktail), such as 100 mg of caffeine (CYP1A2), 25 mg of bupropion (CYP2B6), 25 mg of flurbiprofen (CYP2C9), 5 mg of omeprazole (CYP2C19), 10 mg of dextromethorphan (CYP2D6), 1 mg of midazolam (CYP3A4/5), and 25 mg fexofenadine (P-gp).

The impacts of genetic polymorphisms and different principal covariates and characteristics associated with the disease will be investigated on the activity of measured CYP enzymes comprising gender, BMI, age, blood glucose, and HbA1c levels as secondary endpoints.

In the second phase, the activity of the CYPs mentioned above concerning all other characteristics and covariates will be compared with non-diabetic participants serving as a control group. In other words, any diabetes-related differences in metabolic enzyme activities will be assessed compared to those of the healthy control group, along with the possibility of returning CYP activities to normal levels as a result of good glycemic control.

The research ethics approval was granted by the Tehran University of Medical Sciences Ethics Review Board (Tehran, Iran) (Approval ID: IR.TUMS.TIPS.REC.1397.083). The results of this project will be submitted for publication in a peer-reviewed journal.

### Study population

A total 84 participants, gender-matched between groups, are to be recruited from Diabetes Registry at the Endocrinology and Metabolism outpatient clinic of IMAM KHOMEINI hospital. Study population including a control group of non-type 2 diabetes mellitus subjects and poorly control type 2 diabetic patients will be enrolled in order to provide valuable information to better understand T2D impacts on drug metabolism and to dissect the role of glycaemia versus inflammatory factors.

The present study mainly aimed to include a within-subject comparison to determine whether good glycemic control modulates the phenotype of major CYPs in patients with diabetes. In addition, different groups will be compared to assess any difference in CYP activities before and after glycemic control with healthy participants. In [14], 42 participants with an anticipated dropout rate of 10% leads to sufficient power of study which calculated based on the largest reported variance among all studied activities of CYPs (CYP3A) with 30% reported between-group difference with a power of 80% and an alpha value of 0.05. As a result, the recruitment of an equal number of volunteers with the mentioned study can meet the objectives of this project by considering the elimination of inter-individual differences in the planned study. Therefore, 42 subjects per group will be recruited for a total of 84 participants (public and patients). A stepwise statistical analysis will be performed for each phenotypic probe to determine the modulation effects of both diabetes and glycemic control on CYP activities to cover both phases of the study. In the secondary objective of the present study, the influence of all variables will be examined using multiple regression analyses.

### Public and patient selection and recruitment

Healthy non-diabetic subjects as a control group will be invited to participate in the program through a variety of methods, including face-to-face communication by project researchers, an announcement on the college website, or social media. The patients referred to Diabetes Registry at the Endocrinology and Metabolism outpatient clinic of IMAM KHOMEINI hospital will be scheduled based on clinical indications and be contacted by telephone or in-person at the center by a project researcher. Patients and healthy subjects interested in participation will be informed of the objectives, risks, and inconveniences of the trial. In addition, they will be provided with written information, contact details of the principal investigators as well as copies of their signed consent/assent forms in compliance with Human Ethics guidelines.

All interested participants will be informed of their right to withdraw from the study at any time during the process, without any consequence for their future care. Immediate intervention or treatment is available in case of an acute adverse event, e.g., an anaphylactic reaction. The present protocol does not influence patient treatment procedures.

### Inclusion and exclusion criteria

Healthy adolescent volunteers and patients with type 2 diabetes with inadequate glycemic control defined here as an A1C>7%, aged between 18 or above, body mass index (BMI) ≤ 35**,** without a recent history of hospitalization over the past 12 months will be recruited. The healthy subjects will not have any known history of metabolic diseases, such as diabetes and insulin resistance. All the recruited participants (healthy or diabetic) will not have uncontrolled thyroid problems, cirrhosis, infectious diseases, and IBD. Additional inclusion criteria for all participants are related to being a non-smoker and alcohol abuser. The subjects who are taking medication known to alter metabolism activity, corticosteroids, or NSAIDs with anti-inflammatory dose at least one week before participating in the study will be excluded.

Women will be excluded if they are pregnant or in their breastfeeding stage and will need to have a regular menstrual cycle.

### Experimental protocol and patient follow-up

After the inclusion of poorly controlled patients with diabetes and the public, admitted participants would be invited to the clinical research unit of the hospital. On an experimental day and after an overnight fast, volunteers will be subjected to a 3-hour PK cocktail investigation. All low-dose cocktail capsules will be prepared at the pharmaceutical lab of Pharmacy Faculty under GLP conditions.

Each patient will receive one cocktail capsule containing 25 mg of bupropion, 25 mg of flurbiprofen, 5 mg of omeprazole, 10 mg of dextromethorphan, 1 mg of midazolam, and 25 mg fexofenadine with a cup of coffee (caffeine: 100 mg) and will remain fast until the last time point collection. Four blood samples will be drawn via venous catheters immediately before (time 0), and at 1, 2, and 3 hrs after capsule administration for the probe drugs and their metabolites measurement. The blood samples will be centrifuged immediately after collection at 4 °C and aliquots will then be stored at −80 °C until analysis. Participants will be discharged after the 3-hour sample collection.

Before dosing, two more blood samples will be collected in serum separator SST II tubes and K2-EDTA vacutainers and will be sent to the lab for glucose, HbA1c, and inflammatory marker measurements, respectively.

After the first session of cocktail investigation, patients with diabetes will start a 3-month glycemic control follow-up under the supervision of their physician. During this period, patients will be monitored and motivated by the project researcher for using their prescribed medication. The weight and blood glucose levels of patients will be recorded every month. At the end of this period, cocktail administration and all covariate measurements will be repeated. The healthy volunteers will not undergo any further cocktail investigations or follow-up.

### No patient and public involvement

This research is performed without patient involvement.  Patients are not invited to comment on the study design and were not consulted to develop patient-relevant outcomes or interpret the results. Further, patients are not invited to contribute to the writing or editing of this document for readability or accuracy.

### Laboratory methods

The cocktail probes and their corresponding CYP-specific metabolites will be quantified in plasma using high-performance liquid chromatography/tandem mass spectrometry method, which was previously fully validated and described in [15]. The protein precipitation by acetonitrile will be used for plasma extraction.

Clinical chemistry analysis, including creatinine, AST, ALT, FBS, and HbA1c, will be analyzed, primarily at the Department of Medical Biochemistry at IMAM KHOMEINI hospital complexes.

The plasma levels of proinflammatory cytokines including IL-1β, IL-6, and TNF-α will be quantified by electrochemiluminescence immunoassays using the V-PLEX Proinflammatory Panel 1 Human Kit, QuickPlex SQ120 Imager, and WORKBENCH software (MSD, Rockville, MD). The genetic polymorphism of CYP450 enzymes including CYP2B6, CYP2C9, CYP2C19 and CYP2D6 will be evaluated. Genomic DNA will be extracted from whole blood samples throughout the study in both groups. DNA samples will be used for genetic analyses associated with mentioned CYP450s by using semi-quantitative RT-PCR or other appropriate methods [14,15].

### Data analysis

Pharmacokinetic parameters will be estimated using non-compartmental methods using Excel version 6.2.1. Single-point metabolic ratios (MRs) will be determined as the concentration ratio between the metabolite and the administered probe substrate at different time points. The MRs at the pre-post patient control and patient-healthy phases will be compared using a non-parametric Wilcoxon signed-rank test. Simple and multiple linear regression will be applied to assess correlations between MRs and proinflammatory cytokines as well as other clinical variables. All statistical analyses will be determined using SPSS software version 21 (Chicago, IL). P values ≤ 0.05 will be considered statistically significant.

## Strengths and limitations of this study

### Strengths

• Sampling in a short period (3 h).
• Analyzing the subgroup of different diabetes treatments on the important CYP450 enzyme activity.
• Evaluating the relationship between inflammatory cytokines level and important CYP450 enzyme activity.
• Omitting the inter-individual variations.
• Evaluating the patients’ important CYP450 enzyme genotype to ensure that the changes in CYP450 enzyme activity are due to diabetes and not genetic polymorphisms.

### Limitations

• Following patients for three months.
• Recruiting healthy individuals as a control group.
• Finding diabetic patients that only use insulin for their treatment as one of the subgroups.

## Discussion

The growing prevalence of Type 2 Diabetes (T2D) Mellitus is the most common form of diabetes. T2D is associated with numerous comorbidities and complications in patients due to micro- and macro-vascular problems as a result of elevated blood glucose levels [16,17]. Several inter-individual variabilities are reported in clinical responses to medications, although multiple medications are often required to manage and prevent these issues, [18], [19], [20]. The variability in drug responses may be described partly by modulation effects of diabetes on the functional activity of CYP and P-gp, which may lead to increased risk of treatment failures and ADRs in patients with diabetes [21]. The most important enzymes responsible for phase-I reactions are the monooxygenase and mixed-function oxidase systems. In these reactions, substrate encounters oxidation, reduction, hydrolysis, hydration, and several other reactions [22]. The most important ones with the highest role in xenobiotic metabolisms are CYP1A2, CYP2B6, CYP2C9, CYP2C19, CYP2D6, CYP2E1, and CYP3A4, although cytochrome P450 is divided into various families and subfamilies [23]. So far, several studies have indicated the expression level of cytochrome p450 in T2D changes [8,10]. However, there are few studies on the changes in the activity of these enzymes, specifically in these patients [14]. Most of these studies evaluated the changes in the CYP450 enzyme activity in two groups, i.e., those whose diabetes is well (HbA1c≤7) and not well controlled (HbA1c>7) and carried the risk of inter-individual variations. Further, the effect of different anti-diabetic medicines that patients receive (e.g., insulin or metformin) on the enzyme activity is not evaluated. Another difference between the present study and previous studies is the evaluation of P-gp activity in patients with diabetes [13]. The activity of CYP450 enzymes and P-gp pump in these patients must be understood due to the importance of these enzymes in the metabolism of common drugs and metabolic changes in the development of inter-personal variations. Some studies have been undertaken to evaluate the validity of the cocktail using different criteria to assess the activity of six CYP simultaneously and P-gp isoforms. These studies are performed even with two times lower than the lowest commercialized doses of probes in the administered cocktail, which minimizes the probability of interactions. The further benefits of the cocktail demonstrates using limited blood sampling as phenotyping metrics instead of some blood and urine collections [15,24].

All samples will be analyzed using a single analytical method introduced by the authors after a minimal sample pretreatment that represents a great advantage in terms of cost and time.
